# Supplementary material for: The role of the diencephalon in the guidance of thalamocortical axons in mice
Source: Development. 2020 Jun 26;147(12):dev184523. doi: 10.1242/dev.184523 (PMC7327999; doi:10.1242/dev.184523)
Supplement: Supplementary information [file develop-147-184523-s1.pdf]

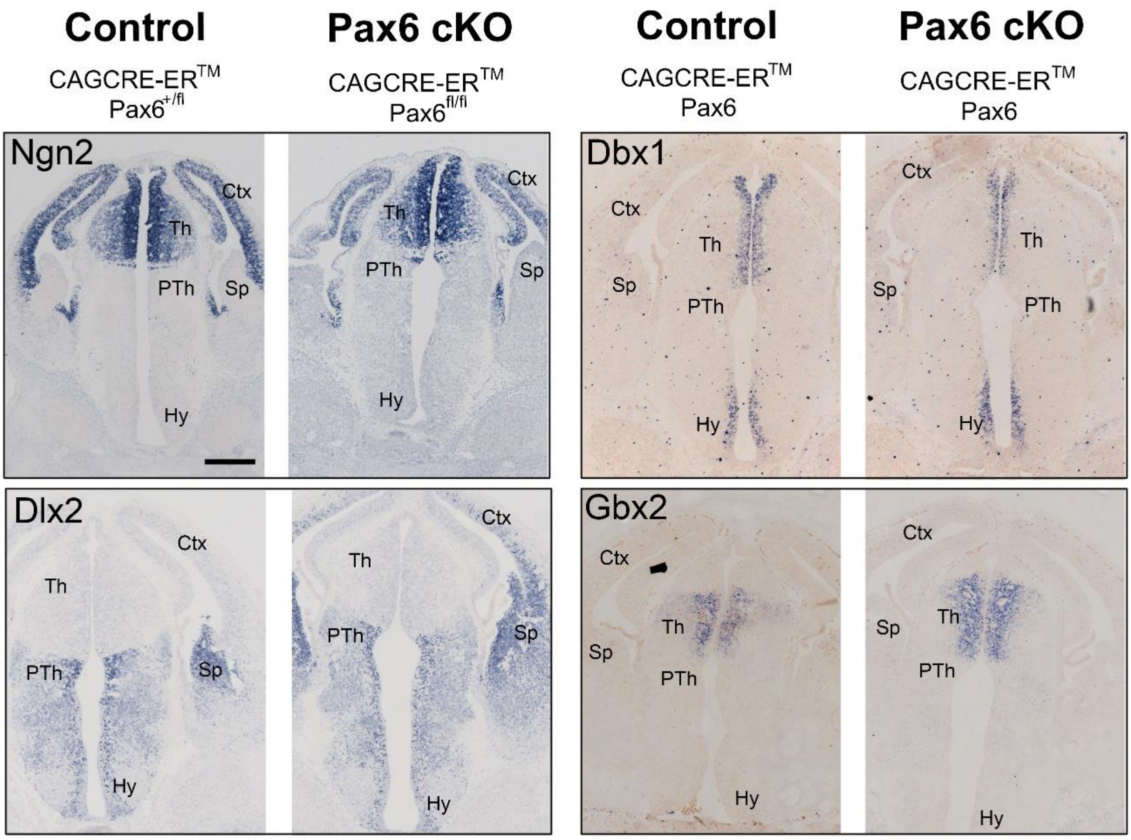

Figure S1. Expression pattern of main transcription factors specifying and delimiting the thalamic progenitor domain are largely unaffected in the thalamus of CAG^{CreER} Pax6 cKOs. Ctx= cortex, Hy= hypothalamus, PTh= prethalamus, Sp= subpallium, Th= thalamus. Scale bar: 500µm

Development • Supplementary information

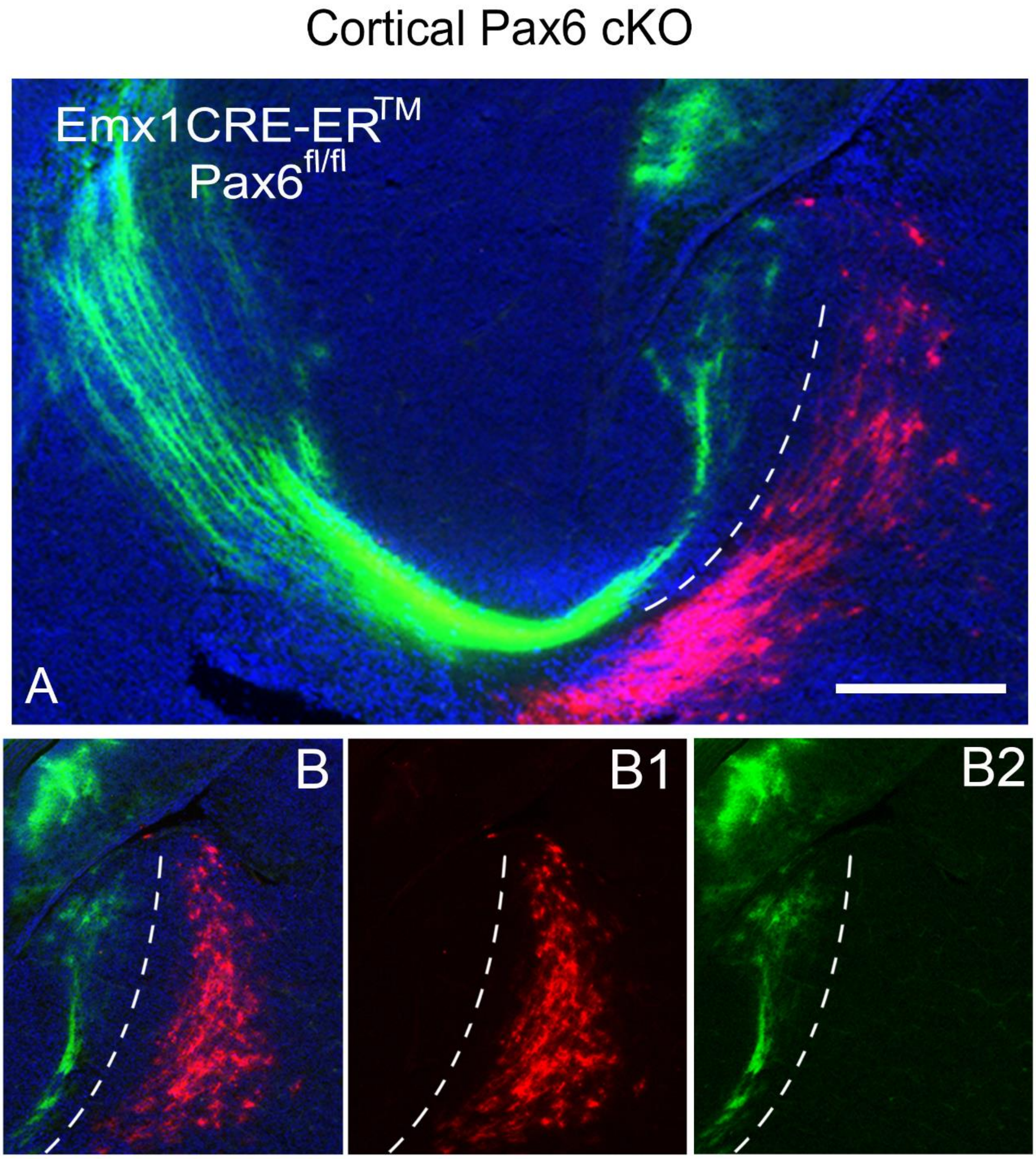

Figure S2. Topographic order is not disrupted in the thalamocortical projection of Emx1Cre^{ERTM} Pax6 cKOs. Panoramic (A) and detail (B) of DiI and DiA labelling in the forebrain of E13.5 Pax6 cortical knockouts. B1 and B2 show separate channels corresponding to image B. Observed in three embryos from three different litters per genotype. Scale bar: 200µm

Development • Supplementary information

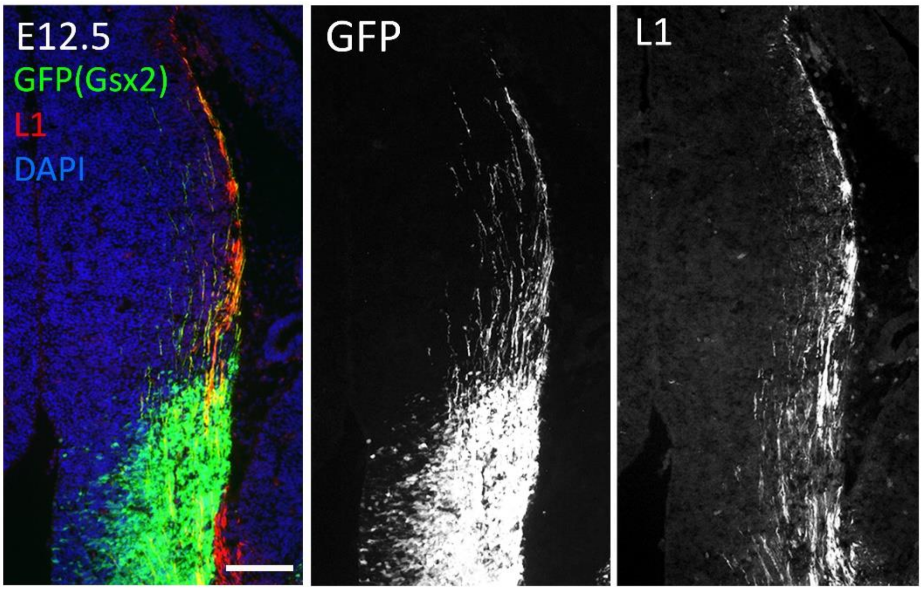

Figure S3. Neurons belonging to the Gsx2 lineage project axons to the thalamus from E12.5. Immunohistochemistry for GFP and L1 showing prethalamic neurons and axons belonging to the Gsx2 lineage (GFP) projecting axons to the thalamus through the same region thalamocortical axons (L1-positive) extend. Scale bar: 100µm

Development • Supplementary information

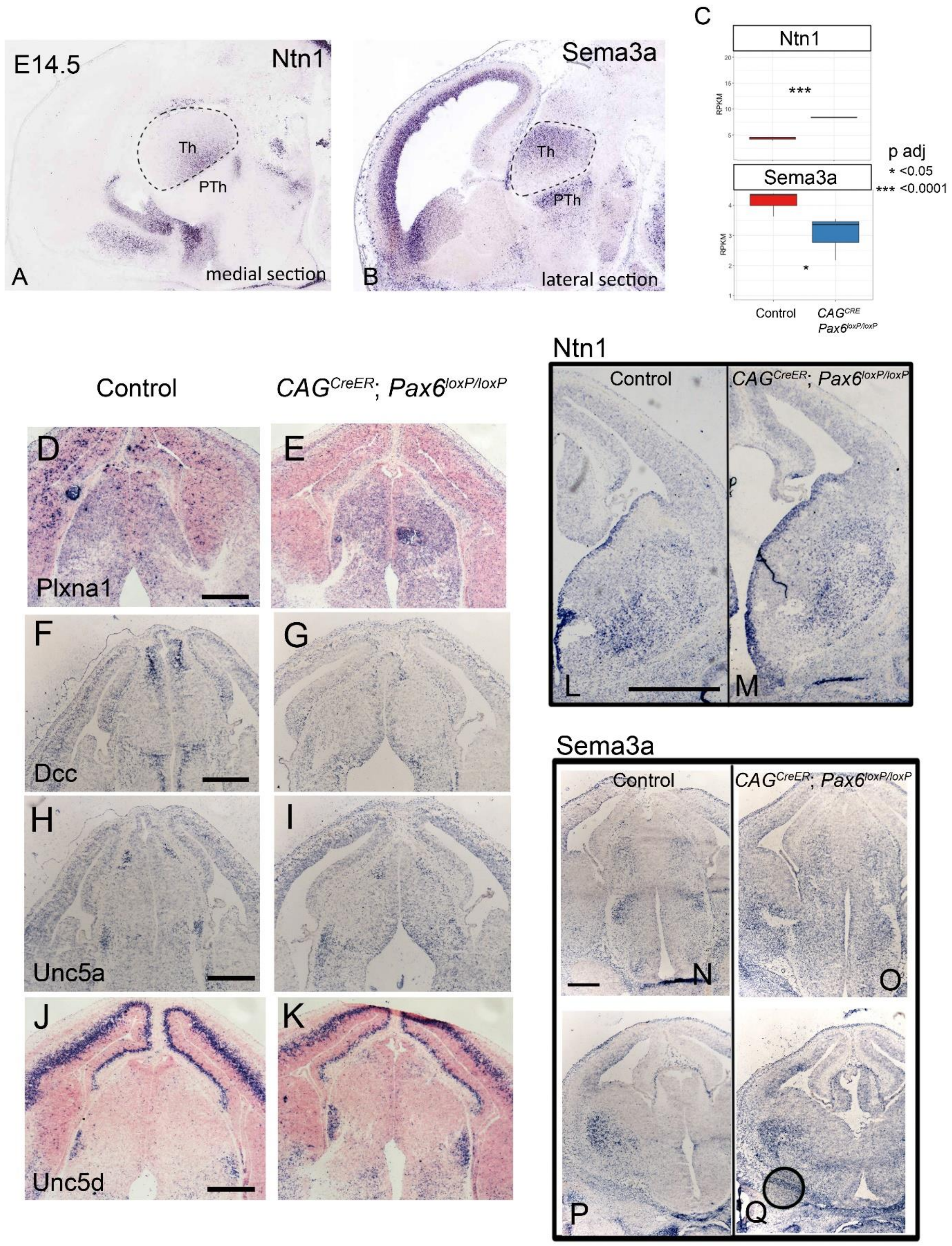

Figure S4. Expression of *Ntn1* and *Sema3a* and some of their receptors in the forebrain of controls and CAG^{CreER} Pax6 cKOs. A,B) Sagittal sections across the

Development • Supplementary information

E14.5 forebrain processed for *in situ* hybridization for *Ntn1* (A) and *Sema3a* (B). Dotted lines indicate the thalamic territory. Note the complementary graded expression pattern between both molecules. Images acquired from GenePaint.org (Visel et al., 2004). **C)** The significance of changes in the expression levels of Sema3a and Ntn1 in the thalamus of CAG^{CreER} Pax6 cKOs was confirmed by analysing a previously published RNAseq dataset from Quintana-Urzainqui et al., 2018**. D-K)** Expression of *Plxna1* (D,E), *Dcc* (F,G), *Unc5a* (H,I) and *Unc5d* (J,K) does not change substantially in the body of the thalamus of E13.5 CAG^{CreER} Pax6 cKOs. **K-N)** Transverse sections of E13.5 telencephalon showing no visible changes in the expression of *Ntn1* (K,L) and *Sema3a* (M,N) in the subpallium of CAG^{CreER} Pax6 cKOs with respect to controls. Scale bars: 500 µm

Development • Supplementary information
