# Supplementary material for: The role of the diencephalon in the guidance of thalamocortical axons in mice
Source: Development. 2020 Jun 26;147(12):dev184523. doi: 10.1242/dev.184523 (PMC7327999; doi:10.1242/dev.184523)
Supplement: Reviewer comments [file develop-147-184523_review_history.pdf]

The role of the diencephalon in the guidance of thalamocortical axons in mice

Idoia Quintana-Urzainqui, Pablo Hernández-Malmierca, James M. Clegg, Ziwen Li, Zrinko Kozić and David J. Price
DOI: 10.1242/dev.184523

Editor: François Guillemot

Review timeline
Original submission: 9 September 2019
Editorial decision: 5 November 2019
First revision received: 10 April 2020
Editorial decision: 6 May 2020
Second revision received: 22 May 2020
Accepted: 2 June 2020

Original submission

First decision letter

MS ID#: DEVELOP/2019/184523

MS TITLE: The role of the diencephalon in the guidance of thalamocortical axons in mice

AUTHORS: Idoia Quintana-Urzainqui, Pablo Hernández-Malmierca, James Clegg, Ziwen Li, and David J. Price

I have now received the reports of three referees on your manuscript and I have reached a decision. The reports are appended below and you can access them online: please go to BenchPress and click on the 'Manuscripts with Decisions' queue in the Author Area.

As you will see, the three referees express great interest in your work, but they also have significant criticisms and recommend a substantial revision of your manuscript before we can consider publication. In particular, they criticise the lack of direct analysis of Pax6 function in the thalamus and they request more quantification of the TCA mistargeting defects. If you are able to revise the manuscript along the lines suggested by the referees, which may involve further experiments, I will be happy to receive a revised version of the manuscript. Some of the experiments requested by referee 2 are beyond what can be achieved during the revision of a manuscript, such as the thalamic-specific deletion of Pax6. However referee 1 suggests an alternative approach of heterotypic explants that seems feasible. Your revised paper will be re-reviewed by the original referees, and its acceptance will depend on your addressing satisfactorily all their major concerns. Please also note that Development will normally permit only one round of major revision.

Please attend to all of the reviewers' comments and ensure that you clearly highlight all changes made in the revised manuscript. Please avoid using 'Tracked changes' in Word files as these are lost in PDF conversion. I should be grateful if you would also provide a point-by-point response detailing how you have dealt with the points raised by the reviewers in the 'Response to Reviewers' box. If you do not agree with any of their criticisms or suggestions please explain clearly why this is so.

© 2020. Published by The Company of Biologists under the terms of the Creative Commons Attribution License (https://creativecommons.org/licenses/by/4.0/).

Reviewer 1

Advance summary and potential significance to field

Although TCA defects in Pax6 mutants have been described for more than two decades, how Pax6 regulates TCA development is difficult to understand because Pax6 is expressed in the thalamus, prethalamus and the cortex at different stages of development. Overall, the authors have used mouse genetics in an elegant way to show convincing data that diencephalic expression of Pax6 plays a role in normal projections of TCAs.

Comments for the author

There are some missing pieces that leave uncertainties as to how Pax6 in diencephalon regulates TCA development.

First, It is unclear if the lack of Pax6 in the thalamus leads to mediolateral mis-targeting of TCAs within the thalamus. Heterotypic slice culture transplants (lateral thalamus of the delayed global Pax6 mutant mice onto wild type thalamus, or vice versa) would solve this problem.

Another issue is that thalamic development of delayed global Pax6 mutant mice (with CAGCreER) is not well described either in this manuscript or in Clegg et al. (2015) paper.
Are progenitor domains intact, unlike the germline Pax6 mutants? Are early postmitotic markers normally expressed but only guidance molecules are altered? Without these data it is hard to fundamentally understand the thalamic phenotypes of these mutant mice.

Reduction of prethalamic axons into the thalamus is shown in Gsx2-Cre driven Pax6 mutants, but not in CAG-CreER driven mutants. Without this, it is not clear if the fasciculation defects in the latter mutant are caused by reduced pioneer axons from the prethalamus. If there is a reduction, then authors also need to consider that changes in expression of guidance molecules in the thalamus are caused by the lack of pioneering axons in these mutant mice, not by lack of Pax6 in the thalamus per se.

Minor points:

1. Images for in situ hybridization are not very crisp. Specifically, Sema3a expression in Fig.6 is blurry and not convincing enough to demonstrate a reduction in Pax6 mutants compared with control mice.

2. In Fig.5F, it is not clear where the telencephalon and the lateral border of the thalamus are.

Reviewer 2

Advance summary and potential significance to field

In this manuscript, the authors explore the roles of Pax6 in the organization of thalamocortical axons (TCAs). In particular, they investigate the roles of Pax6 in the thalamus and pre-thalamus by using temporal inactivation or gsx2-cre driven inactivation. They find that Pax6 temporal inactivation induces a shift in the topography of TCAs as well as a deficit in fasciculation. By knocking out selectively Pax6 in the prethalamus (using the gsx2-cre), they could recapitulate the fasciculation phenotype, but not the defect in topography. The authors show that the fasciculation deficit correlates with a defective extension of axons from the prethalamus. They thus hypothesize that the phenotype observed would be due to a defective axonal tract pathfinding in the thalamus, albeit never directly assess this possibility. To indirectly test this hypothesis, they graft dorsally to the thalamus, thalamic explants and assess the outgrowth of axons, without any quantification. Finally, they examine gene expression patterns in the thalamus of temporal Pax6 cKO and find modifications in the expression of guidance cues and receptors in the thalamus. Based on this collection of observations, the authors conclude that the thalamus contains positional information for TCAs, that the prethalamus regulates the fasciculation of TCAs via axonal guideposts, and that both processes are regulated by Pax6.

© 2020. Published by The Company of Biologists under the terms of the Creative Commons Attribution License (https://creativecommons.org/licenses/by/4.0/).

Understanding the cellular and molecular mechanisms controlling TCA pathfinding is of great interest for our comprehension of axonal tract formation in general and the emergence of a functional neocortex. The topic of this manuscript is thus of interest for a relatively broad readership.
However, in its present form, the paper presents major issues that do not make it suitable for publication in Development.

Comments for the author

In this manuscript, the authors explore the roles of Pax6 in the organization of thalamocortical axons (TCAs). In particular, they investigate the roles of Pax6 in the thalamus and pre-thalamus by using temporal inactivation or gsx2-cre driven inactivation. They find that Pax6 temporal inactivation induces a shift in the topography of TCAs as well as a deficit in fasciculation. By knocking out selectively Pax6 in the prethalamus (using the gsx2-cre), they could recapitulate the fasciculation phenotype, but not the defect in topography. The authors show that the fasciculation deficit correlates with a defective extension of axons from the prethalamus. They thus hypothesize that the phenotype observed would be due to a defective axonal tract pathfinding in the thalamus, albeit never directly assess this possibility. To indirectly test this hypothesis, they graft dorsally to the thalamus, thalamic explants and assess the outgrowth of axons, without any quantification. Finally, they examine gene expression patterns in the thalamus of temporal Pax6 cKO and find modifications in the expression of guidance cues and receptors in the thalamus. Based on this collection of observations, the authors conclude that the thalamus contains positional information for TCAs, that the prethalamus regulates the fasciculation of TCAs via axonal guideposts, and that both processes are regulated by Pax6.

Understanding the cellular and molecular mechanisms controlling TCA pathfinding is of great interest for our comprehension of axonal tract formation in general and the emergence of a functional neocortex. The topic of this manuscript is thus of interest for a relatively broad readership.
However, in its present form, the paper presents major issues that do not make it suitable for publication in Development.

Major Issues

Important claims of the article are not fully supported by the experimental data presented, as detailed below.

1) An important statement is that the comparison between the two cKO mutants (temporal cKO and gsx2-cre cKO) enables the authors to determine the respective roles of Pax6 in the thalamus and prethalamus. However, the pattern of inactivation is never clearly compared throughout the forebrain. In order to conclude that early guidance defects in the diencephalon underlie the cortical mistargeting of TCAs observed in CAG-cre;Pax6cKO mutants, it is essential to show by anterograde tracing that TCAs misroute in the thalamus. In addition, the potential effect of Pax6 deletion from intermediate forebrain structures, such as the ventral telencephalon needs to be ruled out. A detailed timeline of Pax6 deletion CAG-cre;Pax6cKO embryos, compared to the one obtained with gsx2-cre as well as midbrain or vtel specific deletions would help resolve these issues.
2) The authors state that the fasciculation defect that they observe is due to abnormal growth of prethalamic axons. However, these two phenotypes are just correlated and it is impossible to make stronger conclusions with the experimental evidence provided. Similarly, the role of the secreted axon guidance molecules Ntn1, Sema3a and their receptors Unc5c and Plxna1 in early organisation of TCAs within the thalamus is purely correlative. In order to confirm that these pathways are important for TCA guidance in the thalamus – and misexpression underlies the topographic TCA defects observed in CAG-cre;Pax6cKO mutants – guidance of TCAs should be examined in vitro and in vivo using relevant culture and mutant experimental models.

3) Some of the data presented are not fully convincing:
- the pan-neuronal cell adhesion molecule L1 is consistently used throughout the paper as a specific marker of TCAs. L1 is, however, an established axonal marker for other neuronal populations,

© 2020. Published by The Company of Biologists under the terms of the Creative Commons Attribution License (https://creativecommons.org/licenses/by/4.0/).

including corticothalamic axons, dopaminergic axons and potentially prethalamic axons, and therefore does not possess the requisite specificity for TCA organisation.
- the mistargeting of lateral TCAs to more rostral regions of the cortex reported in CAG-cre;Pax6cKO mutants in Figure 1 is difficult to distinguish in the DiI/DiA retrolabelling images presented and remains unconvincing without delineation of the different thalamic structures and robust quantitative analysis. Similarly, in Figures 4 K-L, the reported difference in retrograde labelling of prethalamic neurons by DiI injections in control and CAG-cre;Pax6cKO mutants requires quantification and more detailed annotation.
- the co-culture experiments presented in Figure 5 show in all cases that explants grafted in contact with the pial surface tend to grow superficially. The authors need to provide a clear grafting paradigm as well as provide a quantification of their experiments.
- the in situ hybridization signals presented in Figure 6 (D,D', I, I') show a high lateral expression of Unc5c, which contradicts the schema presented in Figure 6K,L.

Minor comments

1) As presented, there is no clear hypothesis on how temporal inactivation of Pax6 might lead to deficits in thalamic organization, as the gene is not expressed in this structure. I believe that providing even a working hypothesis would greatly improve the manuscript.
2) The figures are difficult to follow in terms of organization and design. It would help the reader to reorganize some of the data as well as provide clear annotations on the pictures.

Reviewer 3

Advance summary and potential significance to field

Using a series of mouse models, the study reports a process of early sorting of thalamocortical axons (TCA) occurring within the thalamus, before their exit towards the pre-thalamus, that is required for proper targeting of their respective final cortical targets. This is an interesting finding that extends our current understanding of the guidepost territories providing positional cues for TCA navigation.

More precisely, the authors first show that Pax6 deletion in the diencephalon generates TCA topographical errors. Second, using mouse conditional mutagenesis to ablate Pax6 in a subpopulation of prethalamic neurons, they provide evidence that the pioneer axons extending from this population, while playing a role in the fasciculation of the TCA is dispensable for the target selection process. Next, they make use of a grafting paradigm in slice cultures to show that cues in the thalamus provide topographical information that sort subsets of TCA along distinct medial and lateral routes. They finally show expression patterns of Netrin1 and Sema3A and some of their known receptors, and elaborate models of their possible contribution to the early medio-lateral organization of TCA tracts in the thalamus.

Comments for the author

General comments
Overall while the evidence that pre-thalamic and thalamic environments bring contribution to the guidance of TCA tracts is compelling, the mechanistic model the authors bring is much weaker because it lacks some important validations. Even if their models are consistent, they are mostly speculative at this stage and also simplistic. For proteins assumed to have localization that largely depends on post-translational events, as it is the case for Netrin1 and likely for sema3A too, in situ hybridization is not ideal to predict how proteins are indeed distributed. A striking example was shown in the recent years for Netrin1 in the developing spinal cord (Varadarajan et al, neuron, 2017). Moreover, to further validate the proposed models, it would be needed to show for example that medial and lateral TCA have distinct responses to Ntn1, in correspondence with their levels of Unc5C, that the navigation of TCAs in slices from Ntn1 -/- and Sema3A mutants is affected, that lateral TCA axons from unc5c-/- behave as those of Pax6 mutants in WT slices….
That said, I also understand that addressing in details these molecular aspects might be largely beyond the scope of the present work. The fact is that the authors almost entirely focus their discussion on the molecular guidance gradients, which gives to the corresponding data a central

© 2020. Published by The Company of Biologists under the terms of the Creative Commons Attribution License (https://creativecommons.org/licenses/by/4.0/).

position in the work. I think that the findings that the thalamus provides early guidance instructions to TCA tracts , that pre-thalamic pioneer axons whose origin is identified in the work also shape the aspect of the TCA tracts are very nice.

The authors could moderate their conclusion on the guidance models, highlight in their discussion the other findings of their work. They could also strengthen some of their data with additional experiments. For example, some experiments to document expression patterns at protein levels could be done, in particular for the receptors. It would be nice to illustrate different expression levels of Unc5c within TCAs. Receptors for Sema3s are Neuropilin/Plexin complexes, the authors should assess Neuropilin1 expression.

More specific comments

1) in Fig1: could the authors provide an estimation of the impact of the misrouting? what is the range of misrouting within the whole population?

2) In Fig 2: It is striking that lateral TCA axons shift their position, which thus concentrates them within a smaller territory. This shift is not quantified. The fasciculation is an active phenomenon which implicates axon-axon recognition. In the end the authors suggest that the mistargeting might result from altered responses of the extracellular guidance cues but here they interpret the changes of TCA organization as a fasciculation phenotype. This would imply that mistargeting results from fasciculation with wrong axons. Do the authors think that this is a wrong initial fasciculation that creates the mistargeting?
But they also have data from Pax6 deletion in Gsx2 prethalamic neurons showing that TCAs can be hyper fasciculated but not misrouted (Fig4). How do they explain this? is this fasciculation phenotype similar to the one induced by Pax6 cdeletion? Is there a positional shift of some TCAs?

3) Do Ntn1 and Sema3A really have a “graded “expression? it looks rather that they are some specific regions of high expression for Ntn1. I can imagine that gradient can arise from protein deposition, but from transcript expression it is not so obvious. I don't see substantial changes of Nnt1 and Sema3A in the Pax mutants…if indeed there are some as claimed by the authors (Ntn1 enlarged, Sema3A reduced) they should be quantified from several embryos.

4) the classical outcome of a release of repulsion is a defasciculation process. Hyperfasciculation is rather linked to increase of exogenous repulsive forces that constrain the axons to grow together. Could the authors comment on how this could fit with their data?

Minor points:

To appreciate the robustness of the data more information is needed on the “number of experiments” that were performed. For example, for the in vivo analyses, how many embryos from how many litters? For the graft-slice assays: how many slices from how many embryos ?, for the expression patterns: how many sections from how many embryos? what means “independent experiments” in these experimental paradigms might be very different.

First revision

Author response to reviewers' comments

REVIEWER 1

“It is unclear if the lack of Pax6 in the thalamus leads to mediolateral mis-targeting of TCAs within the thalamus. Heterotypic slice culture transplants (lateral thalamus of the delayed global Pax6 mutant mice onto wild type thalamus, or vice versa) would solve this problem.”

As the reviewer suggested, we have repeated our heterotypic explant assay but this time using

© 2020. Published by The Company of Biologists under the terms of the Creative Commons Attribution License (https://creativecommons.org/licenses/by/4.0/).

lateral thalamus from CAGCRE^{ER} Pax6 cKO embryos as donor tissue into control thalamic slices. For this, we performed new crossings of our pre-existing lines to generate litters containing GFP-positive Pax6 cKO embryos (CAGCRE$^{ER\ TM}$; Pax6$^{fl/fl}$), and GFP-negative control embryos (homozygous for the Pax6 floxed allele but not expressing the CRE allele). Both embryos carried a RCE:LoxP EGFP allele reporting Cre activity and allowing us to select the embryos within the same litter and visualize Pax6 cKO axons in the explants.

We observed that subsets of axons belonging to Pax6 cKO tissue deviated medially when confronted with control tissue. This is in striking contrast to the invariably lateral trajectories of axons from control lateral thalamic explants grafted into control thalamic tissue (Figure 5). We have quantified new (Figure 7) and previous explants experiments to allow comparison. This further indicates that Pax6 deletion affects the way lateral TCAs navigate through the thalamus, causing some axons to deviate medially and strongly supports our model and hypothesis.

To show these new results, we have added an extra figure (Figure 7), a final section in results (starting in line 370) and relevant information on the experiment in Methods (lines 515,634).

The opposite experiment suggested by the reviewer (GFP-positive control into GFP-negative mutant host) was not possible to achieve in our timeframe. This would involve the generation of new crossings to eliminate the GFP reporter from the mutant line, which will take several months. However, we believe the evidence we provide is enough to prove that Pax6 deletion produces a medial deviation of subsets of lateral TCAs within the thalamus in a cell autonomous manner.

“Another issue is that thalamic development of delayed global Pax6 mutant mice (with CAGCreER) is not well described either in this manuscript or in Clegg et al. (2015) paper. Are progenitor domains intact, unlike the germline Pax6 mutants? Are early postmitotic markers normally expressed but only guidance molecules are altered? Without these data, it is hard to fundamentally understand the thalamic phenotypes of these mutant mice.”

We agree with the reviewer that a description of thalamic development in CAGCRE^{ERTM} Pax6 mutants will benefit the paper. Some aspects of it have been already described in our previous paper where we analysed CAGCRE^{ERTM} Pax6 mutant defects in thalamus, prethalamus and cortex by bulk RNAseq (Quintana-Urzainqui *et al.*, 2018, iScience 10, 171– 191 https://doi.org/10.1016/). Unlike constitutive Pax6 mutants, diencephalic progenitor domains are fully recognizable and relatively intact in our cKOs (see Figures 1 and 3 in Quintana-Urzainqui *et al.*, 2018). In the case of the thalamus, the main effect observed was a decrease in proliferation and increase in differentiation (Quintana-Urzainqui *et al.*, 2018). In fact, thalamic patterning does not seem to be drastically affected. In our hands, the expression of main transcription factors specifying and delimiting the thalamic territory (Ngn2, Gbx2, Dbx1, Dlx2) is not altered in the mutants and we include a new Supplementary Figure to illustrate this (Figure S1). The main transcriptomic changes in the thalamus related with postmitotic neurons corresponded to “Axon Guidance” and “Neuron Projection Development” functional terms (Figure 3 in Quintana-Urzainqui *et al.*, 2018) indicating, as the reviewer mentioned, that postmitotic markers are normally expressed and only guidance molecules seem to be affected. We have added a paragraph in results mentioning this and referring to our previous paper and the new Figure (line 107).

“Reduction of prethalamic axons into the thalamus is shown in Gsx2-Cre driven Pax6 mutants, but not in CAG-CreER driven mutants. Without this, it is not clear if the fasciculation defects in the latter mutant are caused by reduced pioneer axons from the prethalamus. If there is a reduction, then authors also need to consider that changes in expression of guidance molecules in the thalamus are caused by the lack of pioneering axons in these mutant mice, not by lack of Pax6 in the thalamus per se.”

We do not have a genetic tool to label prethalamic axons in the CAG-CRE^{ER} lines, in the way we did for the Gsx2-CRE line, but we presented evidence that prethalamic neurons can be retrogradely traced when injecting DiI in the thalamus in controls but not in CAG-CRE mutants (Fig. 4K,L). This indicated that prethalamic pioneers do not form or do not project to the thalamus in CAG-CRE^{ER} mutants. In any case, the reviewer proposes an interesting question which has never been explored before: are the changes in the expression of guidance molecules a direct result of Pax6 inactivation, or a secondary consequence of the lack of pioneer axon innervation. Although

© 2020. Published by The Company of Biologists under the terms of the Creative Commons Attribution License (https://creativecommons.org/licenses/by/4.0/).

interesting, we think this matter is out of the scope of this manuscript.

"Minor points:
1. Images for in situ hybridization are not very crisp. Specifically, Sema3a expression in Fig.6 is blurry and not convincing enough to demonstrate a reduction in Pax6 mutants compared with control mice."

We agree with the reviewer. These in situs proved very difficult and, unfortunately, we have been unable to improve them. Since our lab is now closed, we have no further possibility of trying to make them look better. We have, therefore, drawn on our previously published RNAseq data allowing comparisons of levels of Sema3a and Ntn1 expression in control vs CAG-CRE^{ER} Pax6 deleted thalamus. These data showed statistically significant downregulation of Sema3a (and upregulation of Ntn1) (dataset published in Quintana- Urzainqui *et al.*, 2018). We have now included specific reference to these findings and the relevant values (line 352 and Figure S4). We hope the referee will agree that together with the quantitative data it is reasonable to say that the pattern of Sema3a expression remains similar to control but the levels are indeed down.
"2. In Fig.5F, it is not clear where the telencephalon and the lateral border of the thalamus are."
We have delineated the structures and added annotation to make this clearer.

REVIEWER 2

"1) *In order to conclude that early guidance defects in the diencephalon underlie the cortical mistargeting of TCAs observed in CAG-cre;Pax6cKO mutants, it is essential to show by anterograde tracing that TCAs misroute in the thalamus. In addition, the potential effect of Pax6 deletion from intermediate forebrain structures, such as the ventral telencephalon needs to be ruled out. A detailed timeline of Pax6 deletion CAG-cre;Pax6cKO embryos, compared to the one obtained with gsx2-cre as well as midbrain or vtel specific deletions would help resolve these issues."*

Regarding the reviewer's first point, that we need to show by anterograde tracing that Pax6 cKO TCAs misroute in the thalamus, we think that new experiments included in response to the reviewers provide the best resolution. We repeated our heterotypic explant assay but this time used lateral thalamus from CAGCRE^{ER} Pax6 cKO embryos as donor tissue into control thalamic slices. For this, we performed new crossings of our pre-existing lines to generate litters containing GFP-positive Pax6 cKO embryos (CAGCRE$^{ER\ TM}$; Pax6$^{fl/fl}$) and GFP- negative control embryos. The GFP in the cKO explants effectively provided anterograde labelling and visualization of Pax6 cKO axons in the diencephalic explants.

We observed subsets of lateral TCAs belonging to Pax6 cKO tissue deviating medially when confronted with control thalamic tissue. We used 11 embryos from four different litters and performed a total of 22 (bilateral) graft experiments. We observed this phenotype in all cases. This is in striking contrast to the invariably lateral trajectories of axons from control lateral thalamic explants grafted into control thalamic tissue (Figure 5). This indicates that Pax6 deletion alters the way lateral TCAs navigate through the thalamus, and that it does that in a cell-autonomous manner.

To show these new results, we have added an extra figure (Figure 7), a final section in results (starting in line 370). Relevant information on the experiment in Methods (lines 515,634). We have quantified new (Figure 7) and previous explants (Figure 5K) experiments to allow comparison. Attempts to anterogradely label TCAs misrouting within the thalamus in CAGCRE Pax6 cKO embryos were less successful. We injected DiI in the E13.5 thalamus of controls and mutants, which anterogradely labelled TCAs (one example is shown in Figure 4K,L). It is very hard to prevent such injections widely labelling much of the thalamic territory making it very difficult to visualize the trajectories of individual axons or bundles within the thalamus.
Attempts at this experiment in earlier embryos (E12.5) proved even more challenging.

On the nature and timing of deletion of Pax6 with the CAGCRE$^{ER\ TM}$ allele, we agree that this is important and it has been described before in Quintana-Urzainqui *et al.*, 2018. We agree that it is difficult to exclude, in the CAGCRE$^{ER\ TM}$ embryos, the possibility of defects of TCA guidance arising due to loss of Pax6 from other extra-diencephalic sites, which is why we think that the new

© 2020. Published by The Company of Biologists under the terms of the Creative Commons Attribution License (https://creativecommons.org/licenses/by/4.0/).

experiments provide the best and most direct test of our interpretation of our findings. We thank the reviewers for suggesting this way forward.

“2) The authors state that the fasciculation defect that they observe is due to abnormal growth of prethalamic axons. However, these two phenotypes are just correlated and it is impossible to make stronger conclusions with the experimental evidence provided.”

We agree our evidence for this is purely correlative. We have acknowledged this in the text and softened the conclusions accordingly (Abstract: line 26, Section title: line 190, line 255).

“Similarly, the role of the secreted axon guidance molecules Ntn1, Sema3a and their receptors Unc5c and Plxna1 in early organisation of TCAs within the thalamus is purely correlative. In order to confirm that these pathways are important for TCA guidance in the thalamus – and misexpression underlies the topographic TCA defects observed in CAG- cre;Pax6cKO mutants – guidance of TCAs should be examined in vitro and in vivo using relevant culture and mutant experimental models.”

We agree that further tests of our molecular model are required to strengthen or refute it. We have done our best to word the manuscript to make clear that this is so, for example “proposing a model” and saying that Ntn1 and Sema3a are “likely” to be involved, and that the patterns “suggest that” our model might have validity. Given the timescale for revision, we prioritized experiments highlighted as important by the journal editor. We would love to do the work the referee suggests but this seems unrealistic in the present context.

“3) Some of the data presented are not fully convincing:
- the pan-neuronal cell adhesion molecule L1 is consistently used throughout the paper as a specific marker of TCAs. L1 is, however, an established axonal marker for other neuronal populations, including corticothalamic axons, dopaminergic axons and potentially prethalamic axons, and therefore does not possess the requisite specificity for TCA organisation.”

We agree with the reviewer that L1 is not a specific marker of TCAs. However, we believe we have not presented it as such. Although not exclusively, it is expressed in thalamocortical axons (Fukuda et al. 1997) and it has proved extremely useful to demarcate TCAs particularly at young ages before corticothalamic axons have fully developed. Here we have used it to study the distribution and bundle width of TCAs in prethalamus at E13.5. At this age corticothalamic axons are not yet in this region (e.g. Nat Rev Neurosci. 2003 Apr;4(4):276-89. Thalamocortical development: how are we going to get there? López- Bendito G1, Molnár Z.), and, in our hands, prethalamic axons do not seem to express L1 (see Fig. 3E

“- the mistargeting of lateral TCAs to more rostral regions of the cortex reported in CAG-cre;Pax6cKO mutants in Figure 1 is difficult to distinguish in the DiI/DiA retrolabelling images presented and remains unconvincing without delineation of the different thalamic structures and robust quantitative analysis.”

Following the reviewer’s advice, we have delineated dLGN and VP nucleus and quantified the percentage of DiI or DiA areas occupying each nucleus in control versus mutants. We found a highly significant increase in the area occupied by DiI in dLGN (values changed from virtually zero to an average of 7.7%). We also found a significant increase of DiA in VP, although the magnitude of this increase was extremely low (0.26%). These results confirm those we described in the earlier version, indicating that a subset of TCAs emerging from dLGN neurons abnormally project to more anterior areas of the cortex. Details of this analysis are included in Results (line 127), Methods (line 619) and the statistical analyses and showed in Figure 1 I .

“Similarly, in Figures 4 K-L, the reported difference in retrograde labelling of prethalamic neurons by DiI injections in control and CAG-cre;Pax6cKO mutants requires quantification and more detailed annotation.”

This phenotype was observed in embryos from three different litters and in all cases we found no retrogradely prethalamic cell bodies in Pax6 cKOs. Therefore, we do not consider quantification necessary. We have annotated the images to delimit the position of the thalamus and prethalamus

© 2020. Published by The Company of Biologists under the terms of the Creative Commons Attribution License (https://creativecommons.org/licenses/by/4.0/).

using as a reference parallel sections immunoreacted for Pax6 (see Figure 4K,L).

"- the co-culture experiments presented in Figure 5 show in all cases that explants grafted in contact with the pial surface tend to grow superficially. The authors need to provide a clear grafting paradigm as well as provide a quantification of their experiments."

We do not think that the reason we observed growth through the lateral thalamus in control grafts is due to the tissue being grafted in contact with the pial surface. First, all grafts were positioned in direct contact with the pre-sectioned interior of the host tissue to avoid pial growth. When we used lateral thalamic tissue as donor (four grafts using embryos from four different litters) we observed lateral trajectories in all cases. Moreover, when grafting lateral thalamus into medial thalamus, we observed axon bundles sharply turning towards very lateral areas, and these axons could not find any pial surface in their trajectory. Additionally, we argue that the fact that medial explants grafted into lateral positions extended axons both lateral and medially (Figure 5J) rules out the possibility that they simply grow along the pial surface.
Second, in our new set of experiments, we performed 22 new grafts of lateral thalamic grafts from Pax6 cKOs, replicating those in Figure 5D. In all cases we observed axons heading in almost all directions, which also rules out the possibility that they simply grow along the pial surface.
We agree with the reviewer that the quantification of these experiments would benefit the paper and we have done so both for previous and new in vitro explants (Fig. 5K, Figure 7C, text:lines 282,380; Methods: 634).

"- the in situ hybridization signals presented in Figure 6 (D,D', I, I') show a high lateral expression of Unc5c, which contradicts the schema presented in Figure 6K,L."

We thank the reviewer for pointing to this potential cause of confusion. We have now added an additional lateral cell to our schemas hoping that we convey the model more clearly.

"Minor comments

1) As presented, there is no clear hypothesis on how temporal inactivation of Pax6 might lead to deficits in thalamic organization, as the gene is not expressed in this structure. I believe that providing even a working hypothesis would greatly improve the manuscript."

Pax6 is expressed in the progenitor neurons of thalamus in a posterior-high anterior-low gradient. In a previous paper, we described deficits in thalamic development in CAGCRE$^{TM\ ER}$ Pax6 cKOs such as a decrease in proliferation and increase in differentiation rates (Quintana-Urzainqui et al., 2018). However thalamic patterning does not appear to be very affected in these mutants. We have now mentioned this paper more clearly in Results (line 107) and added a supplementary figure to illustrate that main transcription factors do not change their expression pattern. Both in our previous paper and in this one we show that the main gene expression changes in postmitotic neurons when Pax6 is deleted in the thalamus are related with axon guidance molecules and axon guidance-related functional terms. Since Pax6 is not expressed in postmitotic neurons we hypothesize that Pax6 expression in progenitors can affect some transcriptional programmes that indirectly translates into actions on the postmitotic expression of certain axon guidance molecules. We have added a paragraph in Discussion referring to this (line 471).

"2) The figures are difficult to follow in terms of organization and design. It would help the reader to reorganize some of the data as well as provide clear annotations on the pictures."

We have added annotation and reorganized some of our figures. We hope this helps to ease the comprehension of the data.

REVIEWER 3

"Overall while the evidence that pre-thalamic and thalamic environments bring contribution to the guidance of TCA tracts is compelling, the mechanistic model the authors bring is much weaker because it lacks some important validations. Even if their models are consistent, they are mostly speculative at this stage and also simplistic. For proteins assumed to have localization that largely

© 2020. Published by The Company of Biologists under the terms of the Creative Commons Attribution License (https://creativecommons.org/licenses/by/4.0/).

depends on post-translational events, as it is the case for Netrin1 and likely for sema3A too, in situ hybridization is not ideal to predict how proteins are indeed distributed. A striking example was shown in the recent years for Netrin1 in the developing spinal cord (Varadarajan et al, neuron, 2017). Moreover, to further validate the proposed models, it would be needed to show for example that medial and lateral TCA have distinct responses to Ntn1, in correspondence with their levels of Unc5C,that the navigation of TCAs in slices from Ntn1 -/- and Sema3A mutants is affected, that lateral TCA axons from unc5c-/- behave as those of Pax6 mutants in WT slices…. That said, I also understand that addressing in details these molecular aspects might be largely beyond the scope of the present work.
The fact is that the authors almost entirely focus their discussion on the molecular guidance gradients, which gives to the corresponding data a central position in the work. I think that the findings that the thalamus provides early guidance instructions to TCA tracts , that pre- thalamic pioneer axons whose origin is identified in the work also shape the aspect of the TCA tracts are very nice. The authors could moderate their conclusion on the guidance models, highlight in their discussion the other findings of their work."

We would not disagree with these views. We think the new experiments in response to the feedback, in which we grafted mutant explants into control thalamus, add a certain amount of strength to existing conclusions but do not get as deep into mechanism as this reviewer suggests one could go. We have modified the discussion, shortening the part dedicated to the model and giving a bit more weight to the rest of our results, as far as we could go respecting the word limit. We thank the reviewer for this suggestion since we really think it balances the manuscript.

"They could also strengthen some of their data with additional experiments. For example, some experiments to document expression patterns at protein levels could be done, in particular for the receptors. It would be nice to illustrate different expression levels of Unc5c within TCAs. Receptors for Sema3s are Neuropilin/Plexin complexes, the authors should assess Neuropilin1 expression."

We agree with the reviewer's thoughts. In practice, we tried protein expression analysis of Sema3a, Ntn1. It proved difficult, probably due to the fact that they are secreted proteins. In fact, we could not find any good antibody in the literature nor good pictures of their expression in the developing forebrain. In our hands, Neuropilin antibody did not work.

"More specific comments

1) in Fig1: could the authors provide an estimation of the impact of the misrouting? what is the range of misrouting within the whole population?"

We have carried out a new quantification. We delimited dLGN and VP nucleus and quantified the percentage of DiI or DiA areas occupying each nucleus in control versus mutants. We found a highly significant increase in the area occupied by DiI in dLGN (values changed from virtually zero to an average of 7.7%). We also found a significant increase of DiA in VP, although the magnitude of this increase was extremely low (0.26%). These results indicate that 7% of labelled TCAs from dLGN misroute towards more anterior cortical targets in CAGCRE Pax6 cKOs. Considering that in controls we have detected virtually 0% of misrouting, we consider this number to be highly significant. Details of this analysis are included in Results (line 127), Methods (line 619) and the statistical analyses and raw data showed in Figure 1 I and Table1, respectively.

"2) In Fig 2: It is striking that lateral TCA axons shift their position, which thus concentrates them within a smaller territory. This shift is not quantified. The fasciculation is an active phenomenon which implicates axon-axon recognition. In the end the authors suggest that the mistargeting might result from altered responses of the extracellular guidance cues but here they interpret the changes of TCA organization as a fasciculation phenotype. This would imply that mistargeting results from fasciculation with wrong axons. Do the authors think that this is a wrong initial fasciculation that creates the mistargeting?
But they also have data from Pax6 deletion in Gsx2 prethalamic neurons showing that TCAs can be hyper fasciculated but not misrouted (Fig4). How do they explain this? is this fasciculation phenotype similar to the one induced by Pax6 cdeletion? Is there a positional shift of some TCAs?"

© 2020. Published by The Company of Biologists under the terms of the Creative Commons Attribution License (https://creativecommons.org/licenses/by/4.0/).

We have thought hard about these comments and we think there might be some confusion. To restate our argument: (1) we think that our evidence shows that there is an abnormality in the way in which TCAs cross the prethalamus, in that they fasciculate abnormally, as the reviewer says; (2) but we think that the fact that this does not cause mapping errors disassociates the fasciculation and the mapping errors. We argue that the fasciculation abnormality does not cause TCA mapping errors. We argue that extracellular guidance cues that are important are those operating in the thalamus itself. So the answer the reviewer's question is that we do not think that "*mistargeting results from fasciculation with wrong axons*". So, exactly as the reviewer says: " *TCAs can be hyper fasciculated but not misrouted (Fig4)"*. This is interesting, we agree, but we don't think there would be any a priori reason to think that abnormal fasciculation would necessarily lead to mistargeting.

"3) Do Ntn1 and Sema3A really have a "graded "expression? it looks rather that they are some specific regions of high expression for Ntn1. I can imagine that gradient can arise from protein deposition, but from transcript expression it is not so obvious. I don't see substantial changes of Nnt1 and Sema3A in the Pax mutants…if indeed there are some as claimed by the authors (Ntn1 enlarged, Sema3A reduced) they should be quantified from several embryos."

We agree that better evidence for these changes in Ntn1 and Sema3A expression is required. We have, therefore, drawn on our previously published RNAseq data allowing comparisons of levels of Sema3a and Ntn1 expression in control vs CAG-CRE^{ER} Pax6 deleted thalamus. These data showed statistically significant downregulation of Sema3a (and upregulation of Ntn1) (Quintana-Urzainqui *et al.*, 2018). We have now included specific reference to these findings and the relevant values (line 352).

"4) the classical outcome of a release of repulsion is a defasciculation process. Hyperfasciculation is rather linked to increase of exogenous repulsive forces that constrain the axons to grow together. Could the authors comment on how this could fit with their data?"

Agreed. We suggest that the hyperfasciculation might be the result of a lack of pioneer axons from the prethalamus that might serve as a scaffold for the growth of small bundles of axons. The presence of these axons might somehow, physically and/or chemically, create a more favourable environment for the growing TCAs. This is very speculative, is based on an association between the fasciculation phenotype and the lack of axons, and does not feed into the main conclusion of the work. Given the strict word limit, it'll be difficult to say more than this in the manuscript.

Minor points:

"To appreciate the robustness of the data more information is needed on the "number of experiments" that were performed. For example, for the in vivo analyses, how many embryos from how many litters? For the graft-slice assays: how many slices from how many embryos ?, for the expression patterns: how many sections from how many embryos? what means "independent experiments" in these experimental paradigms might be very different."

We agree. We have gone through every experiment and clearly stated how many litters, embryos and slices were analysed. For word limit reasons, these data is often included only in the corresponding figure legend and in Methods.

Second decision letter

MS ID#: DEVELOP/2019/184523

MS TITLE: The role of the diencephalon in the guidance of thalamocortical axons in mice

© 2020. Published by The Company of Biologists under the terms of the Creative Commons Attribution License (https://creativecommons.org/licenses/by/4.0/).

AUTHORS: Idoia Quintana-Urzainqui, Pablo Hernández-Malmierca, James Clegg, Ziwen Li, Zrinko Kozic, and David J. Price

I have now received the reports of the three referees who reviewed the earlier version of your manuscript and I have reached a decision. The referees' comments are appended below, or you can access them online: please go to BenchPress and click on the 'Manuscripts with Decisions' queue in the Author Area.

The reviewers' evaluation is overall positive and we would like to publish a revised manuscript in Development, provided that you satisfactorily address the remaining suggestions and comments of referees 1 and 2. You will see that referee 1 criticises the statistical analysis of some of the data, and that referee 2 has several remaining concerns, including the lack of appropriate control for the experiments shown in Figure 7. Please attend to all these comments in your revised manuscript and detail them in your point-by-point response. If you do not agree with any of their criticisms or suggestions, explain clearly why this is so.

We are aware that you may currently be unable to access the lab to undertake experimental revisions. If it would be helpful, we encourage you to contact us to discuss your revision in greater detail. Please send us a point-by-point response indicating where you are able to address concerns raised (either experimentally or by changes to the text) and where you will not be able to do so within the normal timeframe of a revision. We will then provide further guidance. Please also note that we are happy to extend revision timeframes as necessary.

Reviewer 1

Advance summary and potential significance to field

The revised manuscript has been improved by addressing previous comments, and will be an important addition to the work on mechanisms of thalamocortical projections if the inappropriate statistical methods are corrected.

Comments for the author

The authors have now provided appropriate responses to all the comments for the first manuscript. However, I noticed that all the new quantitative data (Fig.1, Fig.5, Fig.7), which respond to other reviewer's comments, suffer from the problem of pseudoreplication, in which the principle of random sampling is violated. The actual number of independent samples should be the same as the number of animals used, not the number of sections analyzed. The authors need to correct this error or argue what they can do without quantification.

Reviewer 2

Advance summary and potential significance to field

Understanding how thalamocortical axons (TCAs), which convey sensory and motor information to tje neocortex, wire up during development is a major question in developmental neuroscience. Here the authors use several conditional Pax6 mutants to show that diencephalic cues regulate the fasciculation of TCAs, but not their topography, whereas borader recombination, including ones in the thalamus perturb topography. The auhtors further describe correlative changes of guidance cues expression and propose that they are important to regulate axonal topography.
The topic is very interesting but the strength of the conclusions put forward not fully supported by the experimental data.

Comments for the author

In this revised manuscript by Quintana-Urzainqui, the authors have attempted addressed the concerns that I raised by adding two novel experiments, performing quantifications, indicating the numbers of experiments performed as well as modifying the text to downtone some claims.

© 2020. Published by The Company of Biologists under the terms of the Creative Commons Attribution License (https://creativecommons.org/licenses/by/4.0/).

While the manuscript has significantly improved, I still believe that the quality of the data does not reach the standards of Development.

Below are presented a few detailed arguments that I believe to be main issues for drawing conclusions and accepting the manuscript as it is for publication

Major Points

1) The main experiment added is presented in Figure 7 - and should be compared to Figure 5 for controls. It consists of grafting lateral mutant thalamic explants into control slices and asssessing their outgrowth. Comparison between Figure 5 (panels C,D) and Figure 7 (panel B), clearly highlight that the width of the thalamus is incomparable in the two experiments (much larger in Figure 5), pointing out that they are not of the same developmental stage. This is likely due to the fact that GFP mice are on swiss/mixed background whereas Pax6cKO are on a different genetic background. In this context, the authors cannot conclude from their experiments on the mutants without having the proper stage- matching controls.

2) Since the authors are not performing any of the experiments requested for the guidance cues, they should at least provide some consistency in what they refer to as lateral path versus medial path. As presented in Figure 6K, thalamic axons, grow laterally, but never superficially on the surface of the diencephalon at E13.5 in vivo (Figure 2A,B,Q- Figure3- Figure 4T), which is what is systematically observed in their explants (Figure 5). This most superficial part is hence not a physiological one ? How does this path relate to the expression of ntn1 and sema3A ? All these issues need to be clarified to provide a consistent model.

3) As stated in my previous review, since the CAG-cre pattern of recombination is not provided, it is difficult to fully establish which struture is accounting for the observed phenotype.

4) Some of the data are still not fully convincing, in particular the reduction in Sema3A expression (Figure 6B compare with Figure 6G) and the fact that Gsx2+ fibers are not positive for L1 (Figure S3 shows some overlapping signal that could be co-expression).

Reviewer 3

Advance summary and potential significance to field

The study reports on novel aspects of the guidance of thalamo-cortical axons.

Comments for the author

In their revised manuscript, the authors provide quantifications of the reported phenotypes, which was one of my major concern. They also add useful technical information for better assessing the number of experiments that were conducted.
I was asking for additional information on expression patterns. These experiments could not be achieved, due to the lack of efficient antibodies. I agree that immunolabeling of secreted proteins is difficult and given the trials made by the authors, their answer is understandable.
Thus, overall, the revisions have strengthened the manuscript and I don't have additional concerns.

Second revision

Author response to reviewers' comments

The revised manuscript has been improved by addressing previous comments, and will be an important addition to the work on mechanisms of thalamocortical projections if the inappropriate

© 2020. Published by The Company of Biologists under the terms of the Creative Commons Attribution License (https://creativecommons.org/licenses/by/4.0/).

statistical methods are corrected.

Reviewer 1 Comments for the Author...

The authors have now provided appropriate responses to all the comments for the first manuscript. However, I noticed that all the new quantitative data (Fig.1, Fig.5, Fig.7), which respond to other reviewer's comments, suffer from the problem of pseudoreplication, in which the principle of random sampling is violated. The actual number of independent samples should be the same as the number of animals used, not the number of sections analyzed. The authors need to correct this error or argue what they can do without quantification.

We thank the reviewer for their kind words.
Regarding the pseudoreplication concerns, we would like to make it clear that we *did* in fact consider each animal or litter as an independent biological replicate, as the reviewer says we should. In our statistical analysis, multiple histological sections from an animal were considered as technical replicates. We believe the confusion might have arisen from a lack of explanation about the statistical method used.
For the statistical analysis we fitted our data to a mixed linear model (line 640), which assesses the biological variation by taking every animal or litter (nested random effects) as a biological replicate to estimate the genotype effect size (fixed effect). In both experiments (Figure 1I, Figures5K+7C) measurements were done on at least five rostral-caudal sections belonging to every biological replicate (embryo or litter, whenever we used more than one embryo per litter). These values were then used to estimate residual errors related to the technical variance and to calculate the significance of genotype effect.
The confusion might have also arisen from the way we plotted the data. In all the figures we present box plots overlapped with dots (Figure 1I, 5K,7C), every dot representing a section value. The reason behind this is to show the actual distribution of the data, in no way meaning that every dot was counted as an independent sample.
The number of litters is indicated in line 134 (figure 1I), line 289-290 (for Figure 5K), and line 382,387 (for Figure 7C). In Methods this is indicated in line 650 (thalamic transplants) and lines 632 (DiI DiA quantification). We have now added explicit mention to what we have considered as biological replicates and clearly stated the N number in Methods section (line 658-664) and figure legends (line 882,964,998).

Reviewer 2 Comments for the Author...

We thank the reviewer for the additional comments. They have led us to clarify some detailed points as explained below and this will surely improve the manuscript.

Major Points

1. The main experiment added is presented in Figure 7 - and should be compared to Figure 5 for controls. It consists of grafting lateral mutant thalamic explants into control slices and asssessing their outgrowth. Comparison between Figure 5 (panels C,D) and Figure 7 (panel B), clearly highlight that the width of the thalamus is incomparable in the two experiments (much larger in Figure 5), pointing out that they are not of the same developmental stage. This is likely due to the fact that GFP mice are on swiss/mixed background whereas Pax6cKO are on a different genetic background. In this context, the authors cannot conclude from their experiments on the mutants without having the proper stage- matching controls.

- Regarding the reviewer’s question about genotypes, the mice used in the two sets of experiments have the same mixed genetic background (CD1, CBA and C67BL/6). We have now added this information to Methods (line 528).

- Regarding the concerns about the size of the embryos:

All embryos used for the slice co-cultures were at E13.5 and then cultured for 3 more days before fixation. There is always some degree of size variation between embryos designated as being of the same gestational age. The reviewer is wondering whether there might be a consistent difference

© 2020. Published by The Company of Biologists under the terms of the Creative Commons Attribution License (https://creativecommons.org/licenses/by/4.0/).

between the sizes of the explants used for experiments in Figure 5 and Figure 7, but we have no evidence for this being the case.
We have prepared low power panoramic pictures of cultures from different litters, which one might expect to show the greatest variation, and show them in a panel at the same magnification in “Figure for reviewers” (cultures shown in the manuscript are included).
Visual inspection of these slice cultures does not reveal consistent size differences of the forebrain as a whole between experiments. There is no indication that they are at different developmental stages. We measured the length of the thalamic ventricular zone and the thickness of the thalamic wall, as shown in these photographs, and again saw no evidence for any consistent differences.
Another important factor to keep in mind is that these are slice cultures that are later cryosectioned. Slight variation in size can also be due to distortion of the tissue in any of these processes. Our method of measurement of levels of GFP from axon outgrowth is, however, relative and not absolute, which would normalize for such variations.
Finally, we would like to point out that we decided to present the new experiments carried out after the initial review in their own Figure and we make no *direct statistical* comparison with the existing data in Figure 5. Our statistical tests in Fig. 7 are done to help assess the pattern of outgrowth from cKO lateral thalamic grafts placed in control lateral thalamus.

We have adjusted the size of Figure 5F and Figure 7B images so they are now at exact same magnification than 5D. We have also added a dotted line marking the midline in Figs 5D and E, hoping this helps to get a more accurate perception of the thickness of the thalamic wall.

Since the authors are not performing any of the experiments requested for the guidance cues, they should at least provide some consistency in what they refer to as lateral path versus medial path. As presented in Figure 6K, thalamic axons, grow laterally, but never superficially on the surface of the diencephalon at E13.5 in vivo (Figure 2A,B,Q- Figure3- Figure 4T), which is what is systematically observed in their explants (Figure 5). This most superficial part is hence not a physiological one ? How does this path relate to the expression of ntn1 and sema3A ? All these issues need to be clarified to provide a consistent model.

What we mean by “lateral” and “medial” thalamic territory is defined in schematics in Fig 5K and 7C. Axons travelling in the lateral-most third of the thalamic thickness = lateral path; axons travelling in the medial-most third of the thalamic thickness = medial path. The reviewer refers to our schematic in Fig. 6K: we think this and other schematics (Fig. 2Q, 4T) did have the potential to confuse. **We have tweaked them to more precisely reflect what we see in the lateral thalamus in vivo.** We now draw the most lateral axon, which is growing in the superficial part of the lateral thalamus, running approximately parallel to the thalamic surface. In Figs. 2Q and 4T, the schematics show axons extending out of the thalamus, and we show the most lateral axons deviating medially as they exit the thalamus and skirt around the laterally placed ventral LG nucleus (vLG). This better reflects the trajectory of these thalamic axons in vivo (e.g. Fig 2A,H; 3A,C; 4N; 5A).

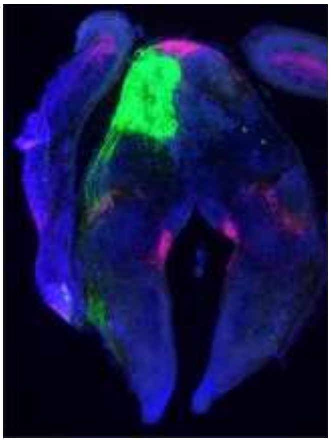

In vitro, as in vivo, lateral thalamic cells send axons through lateral thalamus at a range of distances from the lateral edge of the thalamus (Figure 5D,F in the manuscript, and the picture on the right here shows another example). The main difference in vitro is that the most lateral TCAs

© 2020. Published by The Company of Biologists under the terms of the Creative Commons Attribution License (https://creativecommons.org/licenses/by/4.0/).

do not avoid the vLG as they exit the thalamus and, therefore, do not deviate deeper into the tissue.

To make this point clearer we have now labelled the vLG in Figs 1,2,3, and 5.

We can only speculate that there is something lacking in the culture that allows these axons to grow into the prethalamus without turning to avoid the vLG. This could be the loss of repulsive signals from the vLG? **We find this interesting and have added a sentence in Results to acknowledge it (lines 283-284).** We should also add that, in those cultures in which the link between the diencephalon and telencephalon was maintained, we observed GFP-positive axons crossing the subpallium and heading towards the cortex, indicating that even if they do not show the exact in vivo trajectory of TCAs as they exit the thalamus around the vLG, they do make their way to the telencephalon successfully.

We believe our model, which is focussed on mechanisms *within* the thalamus, still holds as presented in Fig. 6K,L and as explained in the text in (lines 334-345, 369-373, 430-449) and the issues raised here regarding the subsequent trajectory of axons around the vLG will need to be addressed in future work.

3) As stated in my previous review, since the CAG-cre pattern of recombination is not provided, it is difficult to fully establish which struture is accounting for the observed phenotype.

We agree that the pattern of deletion of Pax6 with the CAGCRE^{ERTM} is important. We don't provide it here because it has been described in our previous paper where we analysed CAGCRE^{ERTM} Pax6 mutant defects in thalamus, prethalamus and cortex by bulk RNAseq. After tamoxifen administration at E9.5, no detectable levels of Pax6 were observed from E11.5 onwards across the forebrain (Figure 1 in Quintana-Urzainqui et al., 2018, iScience 10, 171–191 https://doi.org/10.1016/).

We do not have space to reproduce our published findings on the CAG-Cre temporal and spatial patterns of recombination. We believe that the changes in the previous revision should cover this issue: paragraph in Results referring to our previous paper (line 108-115) and the addition of supplementary figure 1 (Fig S1).

We agree that it is difficult to exclude, in the CAGCRE$^{ER\ TM}$ embryos, the possibility of defects of TCA guidance arising due to loss of Pax6 from other extra-diencephalic sites, which is why we think that the new explant experiment provides the best and most direct test of our interpretation of our findings. In our hands, Sema3a and Ntn1 expression pattern in the subpallium appears to be unaffected in CAGCRE Pax6 cKOs (Fig. S4 L-Q).

Our focus in this paper is on testing hypotheses regarding regulatory processes in the diencephalon and not on explaining all consequences of Pax6 loss for TCA guidance at other sites.

4) Some of the data are still not fully convincing, in particular the reduction in Sema3A expression (Figure 6B compare with Figure 6G) and the fact that Gsx2+ fibers are not positive for L1 (Figure S3 shows some overlapping signal that could be co-expression).

Regarding concerns about Sema3a levels, we would like to refer the reviewer to a change made during the previous revision that we hope helps resolve this. Sema3a in situ proved very difficult and, unfortunately, since our lab is closed we had no further possibility to try to improve it. We draw on our previously published RNAseq data allowing comparisons of levels of Sema3a and Ntn1 expression in control vs CAG-CRE^{ER} Pax6 deleted thalamus. These data showed statistically significant downregulation of Sema3a (and upregulation of Ntn1) (dataset published in Quintana-Urzainqui *et al.*, 2018). We included specific reference to these findings and the relevant values in graphs in the current manuscript (line 356-359 and Figure S4C). We hope the referee will agree that together with the quantitative data it is reasonable to say that the pattern of Sema3a expression remains similar to control but the levels are indeed down.

We do not specifically state the fact that Gsx2 fibres are negative for L1. However this could be implied from the phrase in lines 213 "…Gsx2-lineage GFP-positive axons extended throughout the thalamus forming ordered and parallel projections (Fig. 3E) *and running in close apposition to L1-positive TCAs (Fig. 3E',E'')* from E12.5 onwards (Fig. S3)…" . We have now deleted that phrase in italics to avoid confusion.

© 2020. Published by The Company of Biologists under the terms of the Creative Commons Attribution License (https://creativecommons.org/licenses/by/4.0/).

Reviewer 3 Advance Summary and Potential Significance to Field...

The study reports on novel aspects of the guidance of thalamo-cortical axons.

Reviewer 3 Comments for the Author...

In their revised manuscript, the authors provide quantifications of the reported phenotypes, which was one of my major concern. They also add useful technical information for better assessing the number of experiments that were conducted.
I was asking for additional information on expression patterns. These experiments could not be achieved, due to the lack of efficient antibodies. I agree that immunolabeling of secreted proteins is difficult and given the trials made by the authors, their answer is understandable.

We thank the reviewer.

Third decision letter

MS ID#: DEVELOP/2019/184523

MS TITLE: The role of the diencephalon in the guidance of thalamocortical axons in mice

AUTHORS: Idoia Quintana-Urzainqui, Pablo Hernández-Malmierca, James Clegg, Ziwen Li, Zrinko Kozic, and David J. Price
ARTICLE TYPE: Research Article

I am delighted to tell you that your manuscript has been accepted for publication in Development, pending our standard ethics checks.

© 2020. Published by The Company of Biologists under the terms of the Creative Commons Attribution License (https://creativecommons.org/licenses/by/4.0/).
